# Three Discipline Collaborative Radiation Therapy (3DCRT) special debate: Radiation oncology has become so technologically complex that basic fundamental physics should no longer be included in the modern curriculum for radiation oncology residents

**DOI:** 10.1002/acm2.14128

**Published:** 2023-08-22

**Authors:** Sushil Beriwal, Kelsey L. Corrigan, Patrick N. McDermott, Jeffrey Ryckman, May N. Tsao, Dandan Zheng, Michael C. Joiner, Michael M. Dominello, Jay Burmeister

**Affiliations:** ^1^ Department of Radiation Oncology Allegheny Health Network Wexford Pennsylvania USA; ^2^ Department of Radiation Oncology MD Anderson Cancer Center Houston Texas USA; ^3^ Department of Radiation Oncology Corewell Health Royal Oak Michigan USA; ^4^ Camden Clark Comprehensive Regional Cancer Center West Virginia Cancer Institute Parkersburg West Virginia USA; ^5^ Department of Radiation Oncology University of Toronto, Odette Cancer Centre Toronto, ON Canada; ^6^ Department of Radiation Oncology University of Rochester Rochester New York USA; ^7^ Department of Oncology Wayne State University School of Medicine Detroit Michigan USA; ^8^ Gershenson Radiation Oncology Center Barbara Ann Karmanos Cancer Institute Detroit Michigan USA

**Keywords:** radiation oncology resident physics education, resident education

## 1 THREE DISCIPLINE COLLABORATIVE RADIATION THERAPY (3DCRT) DEBATE SERIES

Radiation Oncology is a highly multidisciplinary medical specialty, drawing significantly from three scientific disciplines—medicine, physics, and biology. As a result, discussion of controversies or changes in practice within radiation oncology involves input from all three disciplines. As a result, we have adopted this “team‐science” approach to the traditional debates featured in this journal. This article is part of a series of special debates entitled “Three Discipline Collaborative Radiation Therapy (3DCRT)” in which each debate team has included three multidisciplinary team members, with the hope that this format would be both engaging for the readership and foster further collaboration in the science and clinical practice of radiation oncology. Previous 3DCRT debates have included a radiation oncologist, medical physicist, and radiobiologist on each team. For this debate, we break that trend and include a seasoned radiation oncologist, an early career radiation oncologist, and a medical physicist on each team. We hope these perspectives add valuable insight to this particular debate.

## 2 INTRODUCTION

Physics is one of the fundamental scientific pillars of radiation oncology. Its principles form the foundation for everything from the creation of the radiation we use, to how it interacts in the patient, to how we create and deliver our treatments. As such, it represents one of the core didactic elements of radiation oncology residency training. However, radiation oncology has undergone a staggering increase in technological complexity over the past few decades and our medical residents must be trained to understand and apply these new advances. So how do we add new content to our training curriculum without eliminating or condensing existing content? Medical physics didactic training could be expanded, but this would encroach on other important aspects of training, and these are also expanding. As expressed by Vapiwala in a recent editorial, “keeping up with our field, and oncology in general, is frankly overwhelming”.^1^ If we were instead to eliminate or condense some component of our medical physics didactic curriculum to make room for these new additions, what component would it be? Is it time to retire the most basic and timeworn content? Here we will arbitrarily define “basic fundamental physics” to include the explicit constituents of the “Basic Physics” section of the radiation oncology physics examination blueprint from the American Board of Radiology—“fundamental physics, atomic and nuclear structure, production of kV x‐ray beams, production of MV x‐ray beams, and radiation interactions”. Some would support eliminating this content, arguing that basic fundamental physics is the least “clinically relevant” component and minimizing it would create space for training on current clinical applications of medical physics. After all, when was the last time the factors that determine nuclear stability came up when discussing a patient's treatment in chart rounds? Others would argue that basic fundamental physics is the most important element since it underlies not only all current applications but all new applications that will be developed after the resident has completed training. In other words, “teach your residents about current technology and they will practice effectively for the day, but teach them fundamental physics and they will practice effectively with technological advances for life”. As we continue to expand our training curriculum to produce the most effective future practitioners of radiation oncology, it seems that something may have to give. Should it be basic fundamental physics? This is the subject of this edition of the 3DCRT debate.

Arguing for the proposition will be Drs. Sushil Beriwal, Jeffrey Ryckman, and Dandan Zheng. Dr. Sushil Beriwal worked as a radiation oncologist at UPMC from 2004 to 2021 in various roles, including Residency Program Director, Director of Brachytherapy, and Deputy Director for the UPMC Hillman Cancer Center Network. He has published more than 350 peer‐reviewed articles and book chapters and has received the ABS president award, ARRO teacher of the year, and fellowship from ABS and ASTRO. He is currently Professor of Radiation Oncology at Drexel University, Academic Chief for the Allegheny Health Network, and VP of Medical Affairs for Varian Medical Systems. Dr. Jeff Ryckman is an Assistant Professor at West Virginia University, where he practices as a generalist. He is the chief editor of Rad Onc Review, a free, comprehensive textbook including all aspects of oncology, and the chief content creator for Rad Onc Calc, a free mobile and web application about treating safely. He is also interested in global standardization as evidenced by his involvement with TG‐263U1 working group. Dr. Dandan Zheng is the Director of Medical Physics and Professor in the Department of Radiation Oncology at the University of Rochester. Aside from her involvement in Medical Physics trainee education, she has also been teaching the physics course throughout her career to Radiation Oncology residents at three different universities.

Arguing against the proposition will be Drs. Kelsey L. Corrigan, Patrick N. McDermott, and May N. Tsao. Dr. Kelsey L. Corrigan is a PGY‐5 in radiation oncology at UT MD Anderson Cancer Center who recently passed the American Board of Radiology Physics Board Examination for radiation oncology residents. Dr. Patrick N. McDermott is the Director of Physics Education at Corewell Health (formerly Beaumont Health). He is the coauthor of the textbook “The Physics and Technology of Radiation Therapy,” which was written explicitly for radiation oncology residents. He has been teaching medical residents for 25 years. Dr. May N. Tsao is the Vice Chair of Education and Associate Professor in the Department of Radiation Oncology at the University of Toronto. She, along with her physics colleague Steve Babic, coordinate the yearly applied physics course for the University of Toronto Radiation Oncology and Radiation Physics residents.

## 3 OPENING STATEMENTS

### 3.1 Sushil Beriwal, MD; Jeffrey Ryckman, MD; Dandan Zheng, PhD

If the medical physics course is like a beautiful and intricate garden with carefully arranged and well‐balanced flowers to create a harmonious landscape, adding new flowers without removing any old ones would eventually overwhelm and destroy the garden with the overcrowded flowers competing for space, sunlight, and nutrients. Let's take a look at the new flowers in our field. Over the past few decades, technologies such as IMRT,^2^ SRS,^3^ SGRT,^4^ and particle therapy^5^ have revolutionized our clinical practice landscape. More recently, artificial intelligence (AI) clinical tools are being developed and implemented at an explosive speed,^6^ and new large language model AIs are poised to multiply these efforts even further.^7^ At the same time, our treatment paradigm is rapidly shifting from “what you see on paper is what the patient receives” to evaluating the actual delivery and exploring online adaptive RT^8^ and real‐time motion adaptation.^9^ Innovations like Cherenkov imaging^10^ and PET‐Linac^11^ promise real‐time in‐vivo dosimetry and biologically guided RT. Research directions such as FLASH^12^ and PULSAR^13^ challenge conventional biological and physics models and open new doors. It would be a disservice to the future of our field not to expose radiation oncology residents to these developments or new directions or to prepare them to further these and other endeavors. To create space for the new additions to flourish and prevent our garden from collapsing under its own weight, we must carefully select and remove less relevant flowers. In this debate, we maintain that basic fundamental physics should be removed.

Radiation oncology is a team sport. Medical physics is the domain of the physicist, and radiation oncologists are never without a physicist! Dr. Ryckman (who is on our team) is an MD with a master's degree in Medical Physics, but he confesses that he has never once had to correct his physicist on fundamental physics; instead, he relies on them for those types of questions. If every department has a medical physicist, is it necessary for the physician to “know it all” or just the part that directly influences our practice? If our multidisciplinary practice of radiation oncology is like a Venn diagram, do we have to know the details of every circle or only our own and the parts where our specialties overlap? This is particularly of concern, as the circles keep getting bigger and bigger! Despite the human nature of wanting to be omniscient, the “know it all” expert is unattainable, especially in modern times, as the body of knowledge has evolved so far and become so complex. Today's radiation oncology practice and innovation require a high level of technical expertise and knowledge of complex treatment planning software, advanced imaging modalities, and computer and data science, resulting in a shift towards technology‐driven approaches in radiation oncology. We believe that the field has become so technologically complex that it is no longer necessary for radiation oncology residents to focus their studies on fundamental physics.

Instead, residents need a high‐level understanding of many topics and familiarity with advanced tools and applications for radiation oncology's sustained and expanded future. It is true that physics principles are essential and that a lack of understanding may lead to errors. But the burden of applying fundamental physics for an effective and safe radiation oncology clinical operation rests upon medical physicists instead of radiation oncologists. As fundamental physics rarely comes up in radiation oncologists' clinical discussions with patients, among peers, or with multidisciplinary clinicians, a requirement for its mastery seems very cost‐ineffective in their training. If a fundamental physics question arises, it can be effectively referred to the physician's physicist colleague. For residents, it is more important to understand the limitations of technology than basic physics details. However, important practical training is lacking because currently there is too much emphasis on basic physics in the physics training and testing.

Another critical mission for radiation oncologists is to innovate our field. These innovations will likely come from effective collaborations with medical physicists, biologists, and other experts from diverse disciplines, instead of from radiation oncologists that “know it all”. For residents, the time spent on learning fundamental physics concepts could be better spent on hands‐on training with sophisticated treatment planning and imaging systems, learning new technology and critical topics, clinical research, quality improvement initiatives, and interprofessional collaboration. This way, the residents catch up with the rapidly evolving field and are prepared to propel the next wave of innovation. Even NIH grants are moving increasingly towards multiple‐PI grants than single‐PI grants because, in this day and age, innovations come more from collaborations between experts from different domains than from a single lab or discipline.

In conclusion, subtracting contents is not a sign of weakness or failure but a strategic decision to maintain the integrity and sustainability of the training. Removing basic fundamental physics from the modern curriculum for radiation oncology residents ensures that the remaining important concepts have room to blossom and provide a clear and meaningful path for the residents' learning journey. By omitting the mastery requirement for radiation oncology residents on basic fundamental physics, we can curate a garden of physics knowledge that continues to inspire and engage while avoiding the risk of overwhelming the learners and compromising the overall effectiveness of the training.

### 3.2 Kelsey L. Corrigan, MD; Patrick N. McDermott, PhD; May N. Tsao, MD

Aristotle defined a first principle as “the first basis from which a thing is known”.^14^ These basic fundamentals drive technological advances and are critical to the mastery of any field. As such, having a solid knowledge of basic fundamental physics is critical to the complete understanding of therapeutic radiation. There is a prima facia case against the proposition in this debate. The question should not be whether basic fundamental physics should be taught but what basic physics should be taught. The “staggering increase in technological complexity” argues for an increased emphasis on basic radiation physics. The technology of today will be gone tomorrow. What will remain, however, is basic radiation physics.

Although basic physics is not always obviously applicable in the clinic, it helps the physician understand what we are doing and how we are accomplishing it. These basic fundamental principles will not only guide present practice but will also help with problem solving. Additionally, there are still many clinical scenarios which are based on simple beam planning (e.g., direct orthovoltage, superficial x‐rays, or direct electrons for certain skin cancers). These clinical scenarios require a fundamental knowledge of the differences between these simple beam modalities (e.g., shielding differences, use of bolus or not, how the beam is produced, superficial dose in bone etc.). If we eliminate the requirement to understand basic physics, physicians may lose this fundamental knowledge and could become little more than technicians.

The fundamental building‐blocks of any new technology are basic science and mathematics, and understanding basic physics will help in adopting and understanding more complex radiation technologies. For example, it is only through insight of single photon beam fundamental physics that allows for understanding of multi‐beam arrangements, IMRT, VMAT, SBRT, SRS and how these are produced by specialized radiation machines. The understanding of charged particle physics provides crucial insight into the various proton and heavy ion particle therapy (e.g., helium or carbon) as compared to photons. It is very difficult to say for certain where technology will take us in the future, but basic fundamental physics is the most important element since it underlies not only all current applications but all future applications. As long as radiation remains a therapeutic modality, practitioners will need to know about radiation.

The study of the interaction of radiation with matter is a basic pre‐requisite for radiobiology, which is the basis for the profession of radiation oncology. How can you treat patients with radiation if you don't know what radiation is? Radiation oncologists are writing prescriptions for absorbed radiation dose and are liable for these prescriptions. They should understand what this means. Furthermore, this is applicable to radiation oncologists who practice both in academic medical centers and in the community. In academic medical centers, many research projects in radiation oncology require a sophisticated knowledge of physics. In community treatment centers, radiation oncologists play a vital role for radiation protection and often serve on hospital radiation safety committees. All of these responsibilities require an understanding of radiation and, consequently, the basic physics behind radiation therapy.

Thus, it is a false dichotomy to insist that we must choose to teach either basic physics or new technology—we should continue to teach both. New technology is relatively easy to learn, but basic physics isn't. However, learning them side by side leads to an even better understanding of both, and is applicable and essential for all treating radiation oncologists. We should try to look to the future as best we can and rely on continuing education to keep the practitioner up to date—which will be easiest to accomplish when the individual has a good understanding of the underlying physics and mathematics of radiation.

“I don't know what's the matter with people: they don't learn by understanding; they learn by some other way—by rote or something. Their knowledge is so fragile!”

— Richard Feynman

## 4 REBUTTAL

### 4.1 Sushil Beriwal, MD; Jeffrey Ryckman, MD; Dandan Zheng, PhD

In the opening statement, our opponents quoted Feynman to support their point. It is worth noting that the “Feynman Technique” prioritizes learning through simplicity to build depth of understanding. One might argue that radiation oncology has some of the highest usages of complexity and jargon of any specialty, and our specialty would greatly benefit from teaching physics through the Feynman Technique. Also, given the misinformation regarding radiotherapy in the media, it would help us to train residents who can convey these complex concepts to the general public. Indeed, the fundamental medical physics knowledge needed for residents to safely and effectively practice is straightforward. Still, high‐level radiation physics dominates the first half of dedicated oncology training for busy radiation oncology residents, and mastery is expected.

Fundamental physics displaces other vital topics early in training. The historical era was largely dominated by conventional fractionation and substantially less clinical trial data, but in the modern era, the portion of conventional fractionation keeps getting smaller. We must ask ourselves if we are maintaining all aspects of basic fundamental physics in the curriculum, that it is not as a “rite of passage,” but to prioritize deep understanding of the concepts that drive clinical care on a day‐to‐day basis, especially in the context of all of the other vital information radiation oncology residents are expected to know in the modern era. For instance, one of the most important goals of residency should be to have a deep understanding of plan quality and evaluation so that residents can drive the modernization of clinical practice once they become attending physicians. Still, plan technique/evaluation is often not prioritized until PGY‐4, when the radiation physics board examination has been passed.

Radiation oncology education should focus on mastering advanced tools and principles of radiotherapy planning and advanced image utilization rather than too much emphasis on rote memorization of equations and performing hand calculations, which radiation oncologists hardly use in clinical life. A recent Twitter poll of over 150 radiation oncology participants acknowledged nearly 90% of participants had not done hand calculations in the previous year.^15^ However, rote memorization and hand calculations represent a substantial portion of the resident physics course and board exam. Instead of rote memorization, the focus should be on ensuring residents know where to find the equations and how to perform the calculations in the clinical setting appropriately. The current training introduces fragility into our knowledge base by expecting mastery of basic fundamental physics instead of encouraging understanding of concepts important in day‐to‐day practice.

Our opponents argue that “it is a false dichotomy to insist that we must choose to teach either basic physics or new technology—we should continue to teach both”. In an ideological world, yes, but “know‐it‐all” experts do not exist, or at least not on a mass professional training level. Instead, we have a multidisciplinary team that works together, where we always have medical physicists who we can rely on for solid knowledge of basic physics. In our opponents’ example of “radiation oncologists play a vital role for radiation protection and often serve on hospital radiation safety committees”, their role on these committees does not require solid knowledge of basic physics‐that is the role of the radiation safety officer and the medical physicist on the committee. And that is precisely why we appoint a committee rather than a single person! In the realistic world where time constraints limit the luxury of comprehensive and deep understanding in every subject, radiation oncologists’ role is best played with mastery in their domain, familiarity with modern radiation technology, knowledge of new and future directions, and critical thinking to know where/from whom and what information to locate and also how to interpret and apply that to practice in a multidisciplinary manner.

### 4.2 Kelsey L. Corrigan, MD; Patrick N. McDermott, PhD; May N. Tsao, MD

Fundamentals will always remain relevant. Technical trends will come and go. The simplistic garden analogy fails to recognize that the most beautiful garden relies on older plant species with established roots. The point is to control weeds and sow new seeds, but not to uproot established majestic trees in the process.

We are surprised that our esteemed colleagues state that “basic fundamental physics should be removed”. Radiation oncology is an interdisciplinary specialty, and it is important for each member of the team to have some knowledge of all facets. To become a competent and thoughtful radiation oncologist requires an expert level of understanding of the tools of our trade, namely fundamental physics, such as atomic and nuclear structure, bremsstrahlung x‐ray production, and radiation interactions.

Despite the emergence of new technologies, what has not changed is the basic physics knowledge that is required to understand these technologies. Moreover, these technological changes may be even more confusing for patients. It is the job of the radiation oncologist to not only safely treat patients with these new technologies, but also to explain to patients what is about to happen to their bodies. This is exemplified by these patient questions that are commonly asked during appointments:

“How does radiation work?”

“What kind of radiation am I receiving?”

“What is the difference between external and internal radiation that you are planning for me for my cervical cancer? Why do I need both?”

“Why is my skin cancer being treated with this machine, whereas my cousin's skin cancer was treated with a different machine down the hall?”

“What is the blue light that I see while I am getting treated?”

What happens to the confidence and reassurance regarding treatment safety that patients impart on their radiation oncologists if their treating radiation oncologist replies:

“Sorry I don't know, the field has become so technologically complex, I was not taught nor did I learn that. Let me get my physics colleague to answer all your questions!”

How can you participate in the delivery of “new technology” particle therapy if you do not understand the basic physics of the interactions of protons or heavy ions with matter, and cannot effectively explain this to patients? Our opponents claim that there has been “a shift towards technology‐driven approaches in radiation oncology”. We disagree, technology has always been there from the very beginning, but it has certainly changed! What has not changed is the basic physics knowledge that is required to understand these changes.

To solve the dilemma of adding more information to the physics curriculum, we could remove information regarding old technologies, such as Co‐60 teletherapy units, silver halide radiographic film, conventional simulators, cast blocks, or tissue compensators, which are not used anymore. In other words, remove the “weeds” in the garden analogy. We could also consider removing the requirement to memorize content that could be simply looked up, such as equations, conversion factors, and particle masses. Instead, this information could be provided for residents during their board examination, which would leave space open for new content covering new technologies. This testing method is already commonly done in graduate physics and engineering classes to test students on how they use knowledge instead of unnecessary rote memorization. These examples are logical and feasible strategies for creating space in curricula to introduce new concepts.

In conclusion, we must keep the coverage of relevant basic physics in radiation oncology resident curriculums so that physicians will understand new technology when it comes along, as it surely will. In our garden analogy: new weeds will need to be removed continually, but the foundations on which these new plants are developed will remain the same.

“Teach your residents about technological advances and they will practice effectively for the day, but teach them basic physics and they will practice effectively for life”.

Can't argue with that!

## AUTHOR CONTRIBUTIONS

All authors were responsible for preparation of arguments, and writing and reviewing the manuscript.

## CONFLICT OF INTEREST STATEMENT

The authors declare no conflicts of interest.
